# Perceived coercion to enter treatment among involuntarily and voluntarily admitted patients with substance use disorders

**DOI:** 10.1186/s12913-016-1906-4

**Published:** 2016-11-15

**Authors:** Anne Opsal, Øistein Kristensen, John Kåre Vederhus, Thomas Clausen

**Affiliations:** 1 University of Agder, Faculty of Health and Sport Sciences, Postboks 422, Kristiansand, 4604 Norway; 2 Sørlandet Hospital, Addiction Unit, Postboks 416, Kristiansand, 4604 Norway; 3 Centre for Addiction Research (SERAF), Institute of Clinical medicine, University of Oslo, Postboks 1039 Blindern, Oslo, 0315 Norway

**Keywords:** Substance use disorder, Involuntary admission, Perceived coercion

## Abstract

**Background:**

Perceived coercion is a sense of pressure related to the experience of being referred to treatment. The sense of pressure arises from the patient’s internal perception of coercion. The sources of coercion may be the legal system, the family, the health system, or self-criticism (internal sources). Here, we studied patients diagnosed with substance use disorders that were involuntarily admitted to hospital, pursuant to a social services act. We sought to determine whether these patients perceived coercion differently than patients that were admitted voluntarily.

**Methods:**

This study included patients admitted to combined substance use disorder and psychiatry wards in three publicly funded treatment centres in Norway in the period 2009–2011. Participants included 63 patients that were admitted involuntarily, pursuant to the Norwegian Public Health Act, and 129 patients that were admitted voluntarily. All participants completed the Perceived Coercion Questionnaire. Sociodemographic variables were determined with the European Addiction Severity Index. The range of psychopathological symptoms was evaluated with the Symptom Checklist-90-R. Independent sample t-tests, the chi-squared test, and Fisher’s exact test were used to detect statistically significant differences between groups.

**Results:**

Scores on the Perceived Coercion Questionnaire showed that patients admitted voluntarily and those admitted involuntarily experienced similar levels of perceived coercion. Those admitted voluntarily reported higher levels of perceived coercion from internal sources, and those admitted involuntarily perceived significantly higher coercion from legal sources. No differences between groups were found with the other tests.

**Conclusions:**

Our results suggested that assumptions about involuntary admissions should be evaluated carefully to determine how best to alleviate counterproductive feelings of coercion when a coerced admission is planned. Informing and collaborating with the patient will most likely facilitate a better experience during admission and treatment. Moreover, the patient is more likely to experience a better recovery process.

**Electronic supplementary material:**

The online version of this article (doi:10.1186/s12913-016-1906-4) contains supplementary material, which is available to authorized users.

## Background

Coercion into treatment for substance use disorder (SUD) is practised throughout the world, and it has been the subject of a long-standing ethical debate [1]. According to Klag (2006), critics of legal coercion argue that compulsory treatment may violate basic civil rights [2]. Furthermore, they hold that autonomy should be safeguarded, because free will can provide psychological and therapeutic benefits. Another argument against coercion is that treatment can only be effective when the person is motivated and willing to change. In support of this view, many argue that substance users must hit “rock-bottom” before they are able to recognize that treatment provides benefits. However, others believe that some chronic drug users will not enter and remain in treatment unless they are coerced, and that professionals should have the authority to exercise that power. Still others believe that the authorities should play the role of a surrogate parent, and thus, they have an obligation to intervene on behalf of impaired citizens; this view assumes that, after recovery, patients will be grateful for the intervention [2].

Israelsson found that 73 of 90 countries worldwide provided some form of compulsory commitment (acute or rehabilitative). In all cases, compulsion was motivated by the intent to protect an otherwise legally capable individual in a self-destructive and vulnerable situation, due to substance use [3]. Three main legislative domains, mental health care, social services, and criminal justice, have been described as the foundations for the mandated treatment of patients with SUDs. Civil commitment combines the first two legislative domains [4]. Although most countries may direct one or more of these domains to offer assistance to patients with SUDs, not all countries provide assistance through all three legislative domains. In most cases, involuntary admission of patients with SUDs is a controversial option, which is implemented only after voluntary care has produced unsuccessful results [4–6].

The Norwegian Public Health Act (§10.2) permits involuntary interventions for adult patients with SUDs. The act includes an option to retain the patient for up to 3 months, when voluntary efforts are insufficient, and the health of the patient is seriously at risk, due to extensive, prolonged substance use. In Norway, patients with SUD, whether voluntarily admitted or involuntarily admitted, are often treated in a single ward, and they receive the same types of therapy. In the acute phase, the main goal of retention is to provide life-saving treatment. Over the longer term, the aim is to motivate patients to enter voluntary treatment and engage in long-term recovery [7].

*Perceived coercion* is defined as an individual’s perception of pressure to enter treatment, in this instance for drug and alcohol problems [2]. This pressure may be sensed from sources that are either external or internal to the person. Most previous studies compared various treatment indicators between patients legally mandated to treatment and patients voluntarily committed to treatment. It was previously shown that patients that were admitted voluntarily often experienced informal pressures to enter treatment [8]. Interviews with patients suggested that perceptions of informal coercion were common. The rate of perceived coercion tends to increase with increasing illness severity [9, 10]. Thus, it is simplistic to distinguish patients based only on their formal status (admitted involuntarily or admitted voluntarily), because this classification lacks the complexity of the coercion construct. Furthermore, the formal status classification fails to consider that individuals with drug or alcohol problems are exposed to a wide range of pressures to enter treatment that are not necessarily of a legal nature, including pressures from family, friends, healthcare professionals, or employers [11–13]. From the patient’s point of view, and based on daily law practice, the distinction between voluntary and involuntary admissions may often be ambiguous. Moreover, it is not certain that a legal mandate always causes the patient to feel coerced into treatment or that patients who are self-referred never feel coerced. Due to their condition, some patients may be quite ambivalent about their need for treatment. In other instances, patients may wish to enter voluntary treatment, but do not qualify, or for some reason cannot be offered treatment; then, they are actually relieved to receive mandated treatment [2, 8, 11]. It is important to gain a better understanding of the factors that influence the perceptions of coercion among patients with SUD that are admitted either involuntarily or voluntarily, by comparing these two formally different approaches to treatment. Understanding the coercive forces that contribute to patients entering treatment will provide valuable insight for improving treatment and the overall rehabilitation process. This information also has potential value in addressing multiple issues faced by the treatment provider, the legal system, and policy makers [2].

The present study aimed to investigate the role that perceived coercion played among patients with SUD that entered treatment. We also aimed to clarify whether patients that were admitted involuntarily perceived coercion differently from those that were admitted voluntarily, and to identify factors that could predict perceived coercion.

## Methods

### Study subjects

This study compared two groups of patients admitted to SUD and psychiatry wards: those admitted involuntarily (IA group) and those admitted voluntarily (VA group). Patients in the IA group were recruited from three publicly funded treatment centres in the south-eastern part of Norway. These centres are located in Kristiansand, Tønsberg, and Oslo, and they have four, four, and three beds, respectively, for patients admitted involuntarily. All patients in the VA group were from a single ward in the Kristiansand centre. The three wards are organized quite similarly. They all treat patients of both genders, but most patients were males. The patients may spend time in communal areas, but the exterior doors are locked. Most patients are allowed to leave the ward, when accompanied by a representative from the staff. Many patients received visits from friends and family. Both as a routine procedure and due to suspicion, the patients are required to undergo a urine test for drug-screenings. All wards were multidisciplinary (psychiatrists, psychologists, social workers, occupational therapists, specialized nurses, and other trained staff) and had specialized units that offered treatment for patients with primary SUD combined with mental disorders, a combination which is often observed. Treatment included assessments of somatic and mental health; diagnoses, based on a structured interview and examination, consistent with the International Classification of Diseases and Related Health Problems, 10th Revision (ICD-10); pharmacotherapy; cognitive milieu therapy; and individual motivation enhancement. The patient population was recruited mainly from urban and suburban areas.

Recruitment for the study continued consecutively from 1 January 2009 to 17 December 2011. The criteria for inclusion were: substance abuse or dependence, age ≥18 years, good understanding/speech in Norwegian language, and at least 3 weeks of hospital residence after admission. Approximately 150 decisions are made yearly in Norway concerning the involuntary admission of patients with SUD into institutions, pursuant to the Public Health Act [14].

Before inclusion, patients in both the IA and VA groups were in a detoxified state, verified with negative urine tests for alcohol, opioids, central stimulants (amphetamines, methamphetamines, and cocaine), benzodiazepines, and cannabis. Patients with positive urine tests spent a minimum of 14 days in detoxification to establish baseline values that were not influenced by withdrawal symptoms. Patients were excluded when they exhibited mental retardation (IQ < 70) that prevented them from understanding the questionnaires. Pregnant patients with SUD were treated in specialized wards, and were not included in this study.

We identified 103 consecutive patients that were admitted involuntarily. Among these, 15 did not meet the inclusion criteria (12 patients stayed for an insufficient time period, and 3 patients had insufficient mental capacity); 11 patients were not asked to participate, due to logistical issues. Of the 77 patients eligible for inclusion, 12 patients refused to participate. Therefore, the rate of consent to participate was 84% (65/77 patients). Due to missing scores on the Perceived Coercion Questionnaire (PCQ), two patients were not included in the final analyses. The 63 patients included in the IA group were distributed among three treatment centres: 39 in Kristiansand, 16 in Tønsberg, and 8 in Oslo. We identified 223 patients that were admitted voluntarily; 72 were excluded (69 patients stayed for insufficient time periods, and 3 patients lacked sufficient mental capacity). Of the remaining 151 eligible patients, 14 patients refused to participate. Therefore, the rate of consent in the VA group was 91% (137/151 patients). However, due to missing PCQ scores, eight patients were not included in the final analyses.

The study was approved by The Regional Committee for Research Ethics in Norway (REK 08/206d, 2008/2900, 09/2413) and by the Privacy Issues Unit, Norwegian Social Science Data Services (NSD no. 18782). Written informed consent was obtained from all study participants.

### Instruments and measures

The ICD-10 was used to diagnose current SUDs, the current type and severity of psychiatric problems, and the level of functioning [15]. All patients were interviewed with a clinical psychiatric examination, supported by the Mini International Neuropsychiatric Interview (MINI) version 2005. The MINI is a short psychiatric interview for the assessment of psychiatric disorders, which is in accordance with the Diagnostic and Statistical Manual of Mental Disorders, Fourth Edition and ICD-10 classification systems [16]. This interview has high acceptance and validity [17, 18]. Interviews were conducted by senior psychiatrists and psychologists with several years of clinical and research experience in the psychiatric assessment of patients with physical disorders. Severe SUD was indicated by the injection of illicit drugs within 6 months before admission and a high lifetime frequency of overdoses.

Sociodemographic variables were measured with the European Addiction Severity Index. This personal, structured interview was designed for both clinical and research purposes. It focuses on seven areas: medical status, employment and support status, drug and alcohol use, legal status, family history, family and social relationships, and psychiatric status [19]. Trained and certified staff performed all European Addiction Severity Index-based interviews.

The Symptom Checklist-90-R instrument was used to evaluate the range of psychological problems and symptoms of psychopathology. This test contains 90 items, which measures nine primary symptom dimensions, and it provides an overview of a patient’s symptoms and symptom intensity. Each of the 90 items is rated on a five-point Likert-type scale, ranging from ‘not at all’ (0 points) to ‘extremely’ (4 points); higher values indicate greater symptom severity during the past week. The Global Symptom Index score was used to assess the level of general psychological distress [20].

The PCQ was developed specifically for patients undergoing addiction treatment. The PCQ includes six subscales (Self, Family, Legal, Finance, Health, and Work). Five out of the six subscales measure external coercion to participate in a substance-abuse treatment programme. The sixth subscale, Self, measures an internal form of perceived coercion or pressure. For example, item #1 is, ‘I feel pressure to participate in this drug/alcohol treatment programme…because I know that I’m an addict/alcoholic and that I need rehab to get off drugs/alcohol,’ and #3 is ‘I feel pressure to participate in this drug/alcohol treatment programme…because I feel horrified and ashamed of the person I have turned into’. The PCQ instrument contains 30 items that are presented in the form of statements (see Additional file 1). Respondents rate each statement with a 5-point scale (1, strongly disagree; 2, somewhat disagree; 3, neither agree nor disagree; 4, somewhat agree; 5, strongly agree). A higher score implies a greater degree of coercion perceived by the respondent [2]. Klag et al. (2006) reported that the PCQ has acceptable divergent validity. The validity was demonstrated by a negligible relationship between the PCQ and a presumably unrelated measure (i.e., a measure of spirituality), where the overall *r* was 0.04 and *r* values for PCQ subscales were 0.03–0.10) [2]. A previous analysis of PCQ reliability indicated that the subscales showed adequate internal consistency (Cronbach’s alpha: 0.66–0.87) and good total internal consistency (Cronbach’s alpha: 0.87) [2]. To validate the PCQ in the new setting, we undertook an exploratory factor analysis. First, three items were removed from the PCQ, based on a pre-test of the scale and the notion that these items seemed irrelevant in a Norwegian setting (see Additional file 1). We proceeded to examine whether the remaining items yielded a factor structure similar to that of the original version and whether each item had sufficient factor loadings on its respective subscales. The analysis included principal axis and oblique rotation methods (promax). Kaiser’s eigenvalue-greater-than-one rule was used to determine the number of factors [21]. To ensure internal validity for the present study and to obtain the most parsimonious model, problematic items were removed when they had low factor loadings (<0.4) [21] or did not work as intended. As a result of this analysis, we removed two items in the health subscale. One item was removed, because it represented an extra (seventh) subscale, which constituted a single item only (probably an overfactoring issue: this scale barely exceeded the eigenvalue-of-one criteria). The other item was removed, because it had a low factor loading (see Additional file 1). After these amendments, the scale factors were similar to those in the original scale, and all items had factor loadings > 0.4 on their respective subscales. Based on the PCQ scale results, we divided the patients into two groups: patients that did not perceive any form of coercion and patients that perceived some form of coercion (agreed or strongly agreed) on one or more of the subscales.

### Analysis and statistical methods

Continuous variables are reported as the mean and standard deviation. Categorical variables are reported as the frequency. Independent sample *t*-tests, the chi-squared test, and Fisher’s exact test were used to detect statistically significant differences between groups. The threshold for statistical significance was *p* < 0.05. Linear regression was performed to identify factors that were associated with the PCQ score. Results are presented as β-values with 95% confidence intervals (95% CI) [22]. Analyses were performed with SPSS 22.0 software (SPSS Inc., Chicago, IL, USA).

## Results

### Patient characteristics and substance use

We detected several significant differences between the IA and VA groups (Table 1). All patients met the ICD-10 criteria for current substance dependence or abuse. During the 6 months before admission, significantly more patients in the IA group had injected illicit drugs and at a higher frequency than patients in the VA group. In addition, patients admitted involuntarily had experienced more overdoses during their lifetime compared with patients admitted voluntarily. However, the burden of psychological symptoms (Symptom Checklist-90-R and suicide attempts) was higher in the VA than in the IA group. Significantly more patients admitted involuntarily received ‘no mental diagnoses’, but among all patients with comorbid mental disorders, no significant differences were detected between the IA and VA groups regarding mental health diagnoses. There were significantly more female patients in the IA group than in the VA group (49% vs. 27%, respectively).

**Table 1:** Baseline sociodemographic variables and mental stress scores for patients with substance use disorders

Variable	IA	VA	*P*-value
Age, years, mean (SD)	28.52 (10.6)	30.43 (8.6)	0.177
Female, n (%)	35 (27.1)	31 (49)	0.002
Education			
Years in primary school and high school, mean (SD)	10.58 (1.4)	10.63 (1.6)	0.848
Years in college and university, mean (SD)	0.28 (0.9)	0.18 (0.8)	0.442
Sources of financial support^a, b^			
Employment, n (%)	6 (10)	21 (17)	0.236
Public welfare benefits, n (%)	57 (95)	109 (86)	0.064
Partner, family, or friends, n (%)	17 (29)	37 (30)	0.916
Illegal activity, n (%)	23 (40)	45 (37)	0.691
Living arrangement^b^			
With partner, n (%)	7 (12)	11 (9)	0.512
Alone, n (%)	30 (52)	57 (46)	0.499
With family, n (%)	9 (16)	25 (20)	0.440
No stable arrangements, n (%)	9 (16)	15 (12)	0.539
In a controlled environment, n (%)	2 (3)	15 (12)	0.060
Treated by a physician for somatic diseases^b^, n (%)	23 (43)	27 (23)	0.015
Injecting illicit drug^b^, n (%)	42 (71)	58 (46)	0.001
Alcoholic delirium tremens^c^, n (%)	8 (14)	15 (12)	0.731
Overdoses on drugs^c^, n (%)	40 (70)	61 (50)	0.010
Suicide attempts^c^, n (%)	21 (36)	69 (56)	0.015
Mental stress score			
SCL-90-R GSI, mean (SD)	1.00 (0.7)	1.31 (0.7)	0.004
Number of patients	63	129	

*Abbreviations: IA* involuntarily admitted, *VA* voluntarily admitted, *SD* standard deviation, *SCL-90-R GSI* Symptom Check List-90-Revised, Global Symptom Index

^a^Some participants had more than one source of financial support

^b^Last 6 months before admission

^c^Lifetime prevalence

**Fig. 1:**
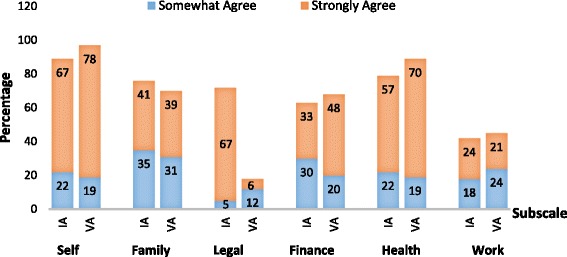
Distribution of the types of coercion perceived by patients with substance use disorders. Patients that were involuntarily or voluntarily admitted for substance abuse treatment completed the Perceived Coercion Questionnaire. Perceived coercion was defined as a report of ‘Somewhat Agree’ or ‘Strongly Agree’ on one or more subscales of the questionnaire. Numbers within the bars indicate the percentage that corresponds to only one coloured portion of the bar. IA: involuntarily admitted group; VA: voluntarily admitted group

The PCQ was used to measure patient perception of external and internal pressures to receive treatment for drug and alcohol problems [2]. Patients in both the IA and VA groups reported perceived coercion during the admission process. When categorizing the PCQ scale, we found that, overall, only 14% of patients admitted involuntarily did not report perceiving coercion on any of the subscales, and 92% of patients admitted voluntarily agreed or strongly agreed that they perceived coercion on one or more of the subscales. The Self and Health subscales reflected the strongest sources of perceived coercion (Fig. 1).

The VA group experienced significantly higher levels of coercion than the IA group in two of the six subscales (Table 2). For example, the mean score for the items on the Self subscale was 0.5 point higher in the VA group (3.3 versus 3.8 for the IA and VA groups, respectively). Closer examination of the Self subscale revealed that the VA group scored higher on three items: ‘Entering this programme is my last and only hope’; ‘I feel horrified and ashamed of the person I have turned into’; and ‘I am sick and tired of losing everything (things, people etc.) because of my drug/alcohol problem’ (Table 3). Notably, only 2/3 of the IA group agreed or strongly agreed with the assertion ‘I felt pressured to enter this drug/alcohol treatment programme, because I was legally required’. Paradoxically, some (12%) of the patients admitted voluntarily agreed or strongly agreed to that same assertion. In the linear regression model, we found that only the Global Score Index of the Scl-90-R was significantly associated with perceived coercion; however, this factor explained only 3% of the variance in the PCQ (Table 4).

**Table 2:** Perceived Coercion Questionnaire (PCQ) scores

Variable	Involuntarily admitted	Voluntarily admitted	*P*-value
Self subscale	16.7 (5.0)	18.9 (4.4)	0.003
Family subscale	15.5 (6.0)	15.0 (6.4)	0.555
Legal subscale^a^	5.6 (2.2)	3.1 (2.1)	0.001
Finance subscale	12.8 (5.5)	13.8 (5.9)	0.259
Health subscale^b^	10.3 (3.5)	11.2 (3.1)	0.076
Work subscale	9.2 (5.2)	10.1 (5.1)	0.256
Total PCQ	76.2 (18.9)	78.0 (18.5)	0.536
Number of patients	63	129	

Values represent the mean (standard deviation)

^a^The Legal subscale of the PCQ underwent minor revisions to account for differences in the Norwegian legal system (see Additional file 1)

^b^The Health subscale of the PCQ has been validated, but it was altered for the present study (see Additional file 1)

**Table 3:** Scores for the Perceived Coercion Questionnaire Self subscale

Self subscale	IA	VA	*P*-value
Self subscale, total score	16.7 (5)	18.9 (4)	0.003
I know that I’m an addict/alcoholic and that I need rehab to get off drugs/alcohol	4.0 (1)	4.3 (1)	0.132
Entering this programme is my last and only hope	2.8 (1)	3.3 (1)	0.023
I don’t know where else to go and what else to do	3.0 (1)	3.4 (1)	0.053
I feel horrified and ashamed of the person I have turned into	3.2 (1)	3.7 (1)	0.013
I am sick and tired of losing everything (things and people) to my drug/alcohol problem	3.7 (1)	4.2 (1)	0.012
Number of patients	63	129	

All values represent the mean (standard deviation); *IA* involuntarily admitted group, *VA* voluntarily admitted group

**Table 4:** Multivariable linear regression analysis results show the effects of independent variables on perceived coercion. N = 192 patients

Variable	Beta (95 % CI)	*P-*value^a^	R^2c^
Female gender	−0.13 (−11.16–1.07)	0.117	
Age	−0.09 (−0.50–0.12)	0.441	
Living alone	−0.01 (−5.80–5.57)	0.713	
Global Score Index: Scl-90-R	0.19 (0.82–9.35)	0.015	3 %
Severity scores			
Injected drug abuse	0.01 (−5.99–6.09)	0.720	
Drug overdoses (lifetime)	0.10 (−2.85–9.99)	0.970	
Suicide attempts (lifetime)	−0.10 (−6.84–5.40)	0.340	
Treatment variable			
Treated for somatic diseases^b^	0.12 (−1.39–11.12)	0.113	
Involuntary hospital admission	−0.03 (−7.90–5.43)	0.536	

^a^ *p*-value obtained from bivariate linear regression. Only one independent variable showed a *p*-value <0.20 in bivariate analyses

^b^During the 6 months prior to admission

^c^ R^2^ adjusted = squared correlation coefficient to obtain a measure of explained variance

## Discussion

This study investigated perceived coercion among patients with SUD that were admitted either involuntarily or voluntarily to receive inpatient hospital treatment. The VA group showed significantly higher scores than the IA group on the internal sources of perceived coercion (Self subscale). No individual characteristics or independent variables were identified that affected perceived coercion in any clinically significant magnitude. Despite the different legal statuses of these groups upon admission to the hospital, the IA group did not report more perceived coercion, overall.

Many clinicians are reluctant to invoke coerced SUD treatment. For some, concern about patient autonomy is the primary deterrence to using coercive treatment, even when the individual’s autonomy is clearly compromised by the cognitive and neurobiological effects of alcohol or substance abuse [23]. In modern bioethics, autonomy is considered one of the overarching ethical principles for protecting patients’ liberties and the right to make their own decisions, for better or worse [24]. Coercion into SUD treatment is commonly equated with a legal mandate. This assumption gives rise to the view that patients referred to treatment by the legal system are coerced, and thus, they must enter against their will. In contrast, individuals that enter without a legal mandate are thought to participate in therapy freely and voluntarily [2]. Our findings supported the view that these assumptions may represent an oversimplification. As expected, patients admitted involuntarily scored significantly higher on the Legal subscale than the patients admitted voluntarily. However, we found that some (14%) patients admitted involuntarily did not report the perception of coercion on any of the PCQ subscales, despite the legal status of their admission. Conversely, 92% of the patients admitted voluntarily agreed or strongly agreed that they perceived coercion, on one or more of the PCQ subscales. Apart from the Legal subscale, we found no significant differences between the IA and VA groups that evidenced greater perceived pressure to enter treatment. However, the VA group reported higher perceived pressure to enter treatment due to internal pressure (Self subscale) than the IA group; this finding indicated that the VA group had greater insight into their own problems, compared to the IA group.

Other studies have suggested that the patients’ experiences of coercion during the admission process in mental hospitals did not necessarily correspond to their legal status [5, 8, 23, 25]. In the present study, we also noted confusion among patients legally required to undergo treatment; 1/3 did not perceive that they were legally required to enter treatment. This misperception of legal status may have been influenced by the fact that, in our study, most of the patients admitted involuntarily stayed in the same wards as the patients admitted voluntarily, and the groups were treated almost equally. Additionally, the Norwegian approach to coercion is largely focused on rehabilitation, motivation, and treatment. Potentially, this premise of the law might influence the attitudes of the professionals involved in this type of treatment. One might speculate that this rehabilitative perspective might be more difficult to find among professionals in countries where coerced measures are guided more often by criminal law and traditions [3].

In coercion studies, it is common to analyse different types of perceived coercion, based on whether the source was external or internal. External coercion is used to motivate the patient to comply with SUD treatment by enforcing alternative consequences, such as a loss of employment or a loss of parental custody. Within the family setting, the consequences of refusing treatment may be broken relationships or the withdrawal of financial or emotional support by family members [23]. An example of an informal type of coercion used to compel a patient to enter treatment is exemplified by the Johnson Intervention. This intervention is a therapeutic technique where the patient’s family or a social group confronts the patient with the consequences of continuous drinking or drug use [23]. This approach is considered coercive, because the family members and friends set forth the consequences of continued drug use, namely certain losses that the individual will face, and these are contrasted with the outcome of SUD treatment. In the present study, both the IA and the VA groups perceived coercion from external sources. The most prominent of the external pressures seemed to arise from family and health-related issues, indicated by the higher scores on these subscales than on the financial and work subscales. The relatively low focus on financial and work issues might be related to the fact that Norwegian health and social welfare systems guarantee at least some level of welfare benefits to patients with SUD conditions. Compared to Klag’s study cohort of Australian patients in residential treatment within a therapeutic community setting, both our patient groups experienced a lesser degree of coercion (Australian vs. IA/VA: Family subscale 18.0 vs. 15.5/15.0; Finance subscale 14.28 vs. 12.8/13.8; and Work subscale 12.37 vs. 9.2/10.1) [2].

The Self subscale of the PCQ consisted of five items intended to measure how internal sources were perceived to coerce individuals into engaging in substance-abuse treatment. Both the IA and VA groups reported higher scores of perceived coercion from internal sources than from external sources, and scores were significantly higher in the VA group than in the IA group. Severe internal pressure (hitting rock bottom) often spurs people into treatment. All the questions on this subscale were related to this concept; consequently, it was not surprising that the internal domains distinguished individuals that sought voluntary treatment from those admitted involuntarily. The authors of the scale noted that “Given the complexity and multitude of pressures that [individuals with SUD] experience, it is reasonable to assume that further sub-categorization of the Self subscale might result in a more reliable and valid measure of the internal pressures that contribute to seeking treatment [2]”. Compared to the Australian patients in residential treatment within a therapeutic community [2], we found that both the IA and VA groups in our study perceived lower levels of coercion from internal sources (Australian vs. IA/VA: Self subscale 21.7 vs. 16.7/18.9).

One argument against invoking the legal coercion act more often in Norway is the notion that patients that perceive coercion would not be motivated to accept treatment. Some researchers hold that, for patients with SUD, the perception of coercion may influence the motivation for treatment, which in turn, was found to influence the patient’s perseverance with treatment and the treatment outcome [26]. Thus, treatment can be effective only when the person is truly motivated, i.e., wants to change. A variation of this position holds that people that are addicted to substances must ‘hit rock bottom’ before they can benefit from treatment [27, 28]; however, this circumstance is not necessarily true for many patients coerced into treatment. According to this view, it is a poor investment to devote resources to patients that are unlikely to change, because they have little or no motivation to change. This argument leads to the empirical question of whether patients that are coerced into treatment lack recognition of their problem, and therefore, have no desire to change [29]. In the present study, we found that the VA group scored higher than the IA group on the Self subscale, indicating that these patients might have had more insight into their own situation. Thus, the VA group could more readily admit and accept that they needed help. Nevertheless, the IA group also scored high on the Self subscale. This suggested that the IA patients also had some insight into their problems. These findings supported the notion that a less motivated patient admitted involuntarily may be able to benefit from the stay on the ward, in ways similar to the benefits observed among the patients admitted voluntarily. When Sullivan compared the Johnson Intervention method of referral to outpatient treatment, the coerced groups were more likely to complete treatment than non-coerced groups [23].

We could not identify any individual characteristics or independent variables that affected perceived coercion in a clinically significant manner, including patient legal status. This result contrasted with findings in other studies that focused on perceived coercion. Previous studies identified male gender, younger age, and illegal drug use as factors associated with higher levels of perceived coercion [2, 30]. This discrepancy might be explained by differences in the criteria for coercion; in Norway, these criteria are very strict; therefore, the selection procedure may have reduced the heterogeneity of the group.

### Limitations and strengths

The findings and conclusions of this analysis must be considered in light of some study limitations. Of note, this study was conducted in a country with a high average income. In addition, due to differences in legislation and practices in different countries and intangible variations in patient expectations and experiences, extrapolations of our findings to other settings are likely to involve complex interpretations. The first limitation was that our data on background characteristics and perceived coercion were self-reported. However, self-reporting should not have a major impact on standard background variables, and it should not affect the ratings of perceived coercion, because they are intended to measure self-perception. Second, the individuals studied in this analysis were selected to represent the general SUD treatment population; however, this sample may also be representative of all Norwegian patients. This patient selection should not differ from patients selected in other countries that apply civil commitment when entering treatment under some form of legal pressure; however, our sample may not necessarily be representative of patients in countries that practice only criminal justice acts. Finally, the relatively small sample size may have limited our power to detect important associations of clinical significance.

The main strength of this study was that it was the first to study experiences of coercion among patients with SUD, despite the fact that this law was established more than 20 years ago in Norway. This study highlighted the patients’ personal views on whether they were forced to undergo SUD treatment, irrespective of their legal status. This type of research is important because it sheds light on the processes that patients with SUD undergo, when the society invokes coercive laws with a rehabilitative purpose. More nuanced research is needed to understand how the patient’s perception of coercion is related to the motivation to enter treatment. An important topic for future research is the relationship between objective and perceived coercion.

### Methodological considerations

Although the PCQ is considered a valid scale for the present setting, our amendments of the scale preclude comparison with other studies, both for the total PCQ score and for the altered subscales. However, the present study did not aim to conduct a cross-cultural comparison; therefore, we chose to focus on internal validity in the present setting.

## Conclusion

Patients in both the IA and VA groups perceived some level of coercion to enter into treatment for SUDs. The VA and IA groups experienced similar overall levels of perceived coercion, but the VA group reported higher levels of perceived coercion from internal sources (Self subscale). As expected, the IA group scored significantly higher on the Legal subscale. All patients with SUD, whether involuntarily or voluntarily admitted, experienced high levels of perceived coercion that was unrelated to the law. Internal sources of perceived coercion were dominant in both groups.

Understanding the coercive forces that compel patients to enter treatment will provide valuable insight into ways to improve treatment and the overall process of patient rehabilitation. The information obtained here has potential value in addressing multiple issues for the treatment provider, the legal system, and policy makers, alike. For example, for therapists, a better understanding is important in treatment planning and in monitoring patient progress. Patients that feel pressured to be in treatment by external sources could receive interventions that reduce those pressures; this intervention might thereby increase the patient’s motivation to engage in treatment. Therefore, we anticipate that treatment providers might find it useful to implement the PCQ as part of their assessment.

Every clinical encounter includes a potential element of coercion and hierarchy; these components may be inescapable in any relationship between patients and healthcare personnel. However, knowledge of factors that lead to perceived coercion may elucidate ways to limit these components. The observations presented in this study indicated that, when a coerced admission is planned, information and collaboration with the patient will likely facilitate a better experience during admission and treatment for the patients, and thereby, a better process towards recovery. The findings of this study may yield new insights for healthcare professionals and policy makers.

## Additional file

Additional file 1: 30-Item Perceived Coercion Questionnaire. Description: List of questions in the Perceived Coercion Questionnaire, and the alterations applied for the present study. (DOCX 90 kb)

## Abbreviations

IA: Involuntarily admitted
PCQ: Perceived Coercion Questionnaire
SUD: Substance use disorders
VA: Voluntarily admitted
