# Supplementary material for: Perceived coercion to enter treatment among involuntarily and voluntarily admitted patients with substance use disorders
Source: BMC Health Serv Res. 2016 Nov 15;16:656. doi: 10.1186/s12913-016-1906-4 (PMC5111249; doi:10.1186/s12913-016-1906-4)
Supplement: Additional file 1: — 30-Item Perceived Coercion Questionnaire. Description: List of questions in the Perceived Coercion Questionnaire, and the alterations applied for the present study. (DOCX 90 kb) [file 12913_2016_1906_MOESM1_ESM.docx]

**Additional file 1**

**30-Item Perceived Coercion Questionnaire**

**I felt pressured to enter this drug/alcohol treatment program because…**

1. I know that I’m an addict/alcoholic and that I need rehab to get off drugs/alcohol

2. Entering this program is my last and only hope

3. I don’t know where else to go and what else to do

4. I feel horrified and ashamed of the person I have turned into

5. My family kept telling me what a bad person I have turned into and how messed up I am

6. I’m sick and tired of losing everything (e.g., things, people etc.) to drug/alcohol problem

10. Members of my family got upset all the time about my drug/alcohol use (crying, screaming etc.)

11. I was legally required

12. I had the choice between jail and rehab

*13. People from the legal system (e.g., police) kept knocking on my door, threatening me with jail

*14. People in the legal system forced me to do this

*15. I didn’t want to do time in jail

16. I’m up to my ears in financial problems and don’t know what to do

17. I wanted to escape people/institutions that are after me for money

18. I have lost all financial support (e.g., from family, banks, government departments etc.) 19. I have no money to support myself

20. I’m sick and tired of being in debt

21. of my bad health

22. I have had enough of being sick all the time

23. I am scared of the negative consequences on my health if I don’t stop using/drinking

**24. Health care professionals (e.g., doctors, psychologists etc.) kept telling me that I need rehab

**25. People kept telling me how sick I look

26. I lost my job due to my substance use

27. My employer threatened to sack me

28. My colleagues at work kept telling me that I need rehab

29. My work colleagues complained about me

30. I got into a lot of trouble at work because of my substance abuse

* Questions 13, 14, and 15 were removed from the Legal subscale, because the Norwegian law is different from the Australian laws, where the PCQ was developed. **Questions 24 and 25 were removed to obtain internal validity.
